# TGF-β1 stimulates migration of type II endometrial cancer cells by down-regulating PTEN via activation of SMAD and ERK1/2 signaling pathways

**DOI:** 10.18632/oncotarget.11311

**Published:** 2016-08-16

**Authors:** Siyuan Xiong, Jung-Chien Cheng, Christian Klausen, Jianfang Zhao, Peter C.K. Leung

**Affiliations:** ^1^ Department of Obstetrics and Gynaecology, Child and Family Research Institute, University of British Columbia, Vancouver, British Columbia, Canada V5Z 4H4

**Keywords:** TGF-β1, PTEN, type II endometrial cancer, migration

## Abstract

PTEN acts as a tumor suppressor primarily by antagonizing the PI3K/AKT signaling pathway. *PTEN* is frequently mutated in human cancers; however, in type II endometrial cancers its mutation rate is very low. Overexpression of TGF-β1 and its receptors has been reported to correlate with metastasis of human cancers and reduced survival rates. Although TGF-β1 has been shown to regulate PTEN expression through various mechanisms, it is not yet known if the same is true in type II endometrial cancer. In the present study, we show that treatment with TGF-β1 stimulates the migration of two type II endometrial cancer cell lines, KLE and HEC-50. In addition, TGF-β1 treatment down-regulates both mRNA and protein levels of PTEN. Overexpression of PTEN or inhibition of PI3K abolishes TGF-β1-stimulated cell migration. TGF-β1 induces SMAD2/3 phosphorylation and knockdown of common SMAD4 inhibits the suppressive effects of TGF-β1 on PTEN mRNA and protein. Interestingly, TGF-β1 induces ERK1/2 phosphorylation and pre-treatment with a MEK inhibitor attenuates the suppression of PTEN protein, but not mRNA, by TGF-β1. This study provides important insights into the molecular mechanisms mediating TGF-β1-induced down-regulation of PTEN and demonstrates an important role of PTEN in the regulation of type II endometrial cancer cell migration.

## INTRODUCTION

In addition to being the fourth most commonly diagnosed female malignancy in North America, endometrial cancer is the most common, and second most lethal, gynecological cancer [1]. Endometrial cancers are often classified into two general clinicopathological types [2]. Type I endometrial tumors, which account for ~70% of endometrial cancers, are primarily comprised of low-grade endometrioid tumors and are associated with favorable prognosis. Type II endometrial cancers comprise a group of high-risk tumors of serous, clear cell or high-grade endometrioid histology that are highly invasive and associated with poor survival [3, 4]. Indeed, approximately 75% of endometrial cancer related deaths can be attributed to the aggressive behaviour of these high-risk tumors [3–5]. Investigations into the molecular mechanisms contributing to type II endometrial cancer metastasis could provide insight for the development of improved therapeutic strategies.

TGF-β1 belongs to the TGF-β superfamily which regulates a wide array of biological functions, including cell proliferation, differentiation, migration and apoptosis [6]. Overexpression of TGF-β1 has been reported in several human cancers and correlates with metastasis and reduced survival rates. Furthermore, its immunoreactivity is stronger in invasive lymph node metastases than primary tumor sites [7]. TGF-β1 thus serves as a biomarker for poor prognosis and a potential therapeutic target in such malignancies [8, 9]. TGF-β1 mRNA, protein and receptors are expressed in normal and neoplastic human endometrial tissues or endometrial cancer cell lines [10–13]. Moreover, immunohistochemical studies suggest that the expression of TGF-β1 is increased in the epithelial component of endometrial carcinomas compared with non-neoplastic tissues [10, 12], and the plasma level of TGF-β1 is greater in Stage-Ib and Stage-Ic patients than in Stage-Ia patients [14]. These results suggest that TGF-β1 may act in an autocrine and/or paracrine manner to regulate important aspects of endometrial cancer biology. Although early studies demonstrated either inhibitory or no effects of TGF-β1 on the growth of endometrial cancer cell lines [13, 15, 16], recent gene expression profiling studies suggest the TGF-β1 gene network may contribute to an elevated risk of recurrence [17]. Moreover, TGF-β1 has been shown to enhance the invasiveness of HEC-1A and RL95-2 human endometrial cancer cells [17, 18]. These findings suggest that TGF-β1 exerts its tumor-promoting roles in human endometrial cancer mainly through the support of metastasis.

PTEN (phosphatase and tensin homologue) is a well-known and frequently mutated tumor suppressor that antagonizes the phosphoinositide 3-kinase (PI3K)/AKT signaling pathway. Loss of PTEN function causes hyperactivation of the PI3K/AKT pathway, altering cell growth, apoptosis, invasion and metastasis [19]. Studies suggest that 50–80% of type I endometrial cancers harbour PTEN genetic mutations, whereas only 10% of type II endometrial tumors have mutant PTEN [3, 20]. Interestingly, the rate of PTEN protein loss in type II endometrial cancer is much higher than alterations in the *PTEN* gene [21, 22]. These findings indicate that PTEN expression can be reduced or lost via transcriptional or post-translational mechanisms. Indeed, treatment with TGF-β1 has been shown to down-regulate PTEN protein levels by increasing its degradation in KLE human type II endometrial cancer cells [23]. However, the degree to which PTEN expression can be transcriptionally regulated by TGF-β1 in human type II endometrial cancer cells and the mechanisms underlying this potential mode of regulation remain unclear.

In the present study, we show that TGF-β1 stimulates the migration of KLE and HEC-50 type II endometrial cancer cell lines. Additionally, we show that both mRNA and protein levels of PTEN are down-regulated by TGF-β1 treatment. Overexpression of PTEN and inhibition of the PI3K/AKT pathway abolished the effects of TGF-β1 on cell migration. Interestingly, we show that the SMAD2/3-SMAD4 and MEK-ERK1/2 pathways are differentially involved in the down-regulation of PTEN mRNA and protein by TGF-β1. Our findings indicate that PTEN may act as an important mediator in TGF-β1-stimulated type II endometrial cancer cell migration.

## RESULTS

### TGF-β1 increases the migration of type II endometrial cancer cells

**Figure 1:**
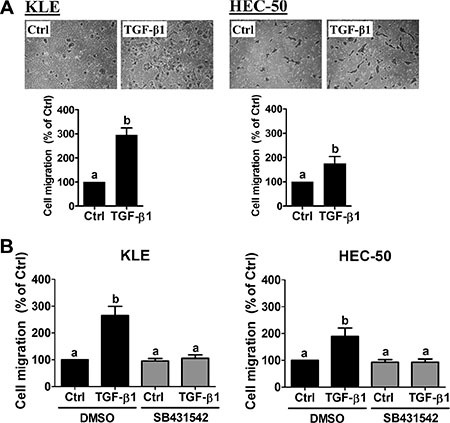
TGF-β1 stimulates type II endometrial cancer cell migration (**A**) KLE and HEC-50 cells were treated without (Ctrl) or with 10 ng/mL TGF-β1 for 24 h and then seeded into transwell inserts for the 24-hour migration assay. Upper panels show representative photomicrographs of migrating cells, while lower panels show summarized quantitative results. (**B**) KLE and HEC-50 cells were pre-treated with vehicle (DMSO) or SB431542 (10 μM) for 1 h and then treated with 10 ng/mL TGF-β1 for 24 h. After treatment, the levels of cell migration were examined by the transwell migration assay (24 h). Results are expressed as the mean ± SEM of at least three independent experiments and values without common letters are significantly different (*P* < 0.05).

The survival rate of endometrial cancer drops from 90% to less than 17% once invasion and metastasis occur [1, 24]. Therefore, we first investigated the effect of TGF-β1 on cell migration in two type II endometrial cancer cell lines, KLE and HEC-50. Boyden chamber transwell migration assays revealed that treatment with 10 ng/mL TGF-β1 significantly increased cell migration in both cell lines (Figure 1A). Moreover, the stimulatory effects of TGF-β1 on cell migration were abolished by pre-treatment with the specific TGF-β type I receptor inhibitor SB431542 (Figure 1B). These results suggest that TGF-β1 acts via TGF-β type I receptor to increase type II endometrial cancer cell migration.

### TGF-β1 down-regulates PTEN in type II endometrial cancer cells

**Figure 2:**
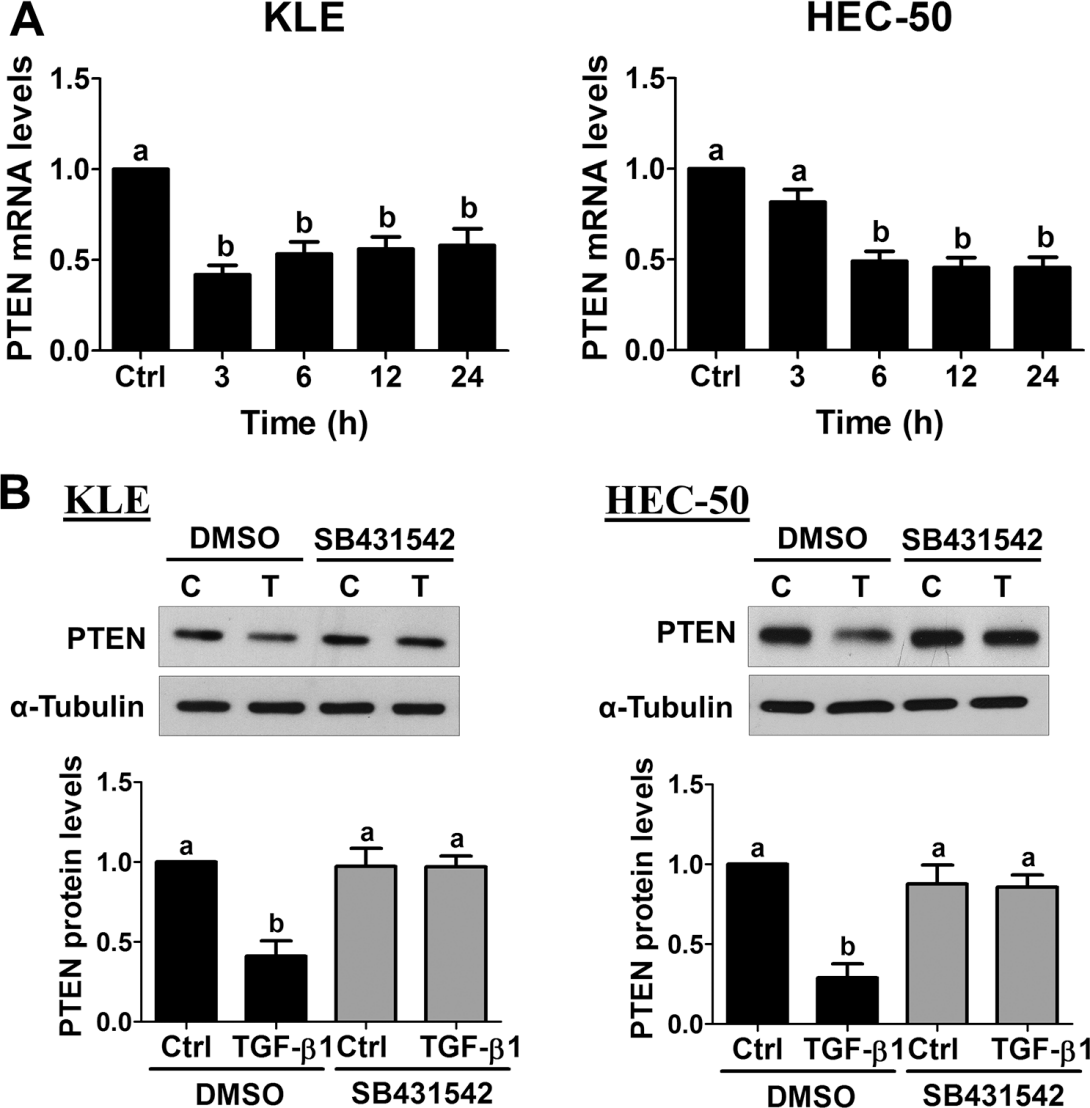
TGF-β1 down-regulates PTEN expression in type II endometrial cancer cells (**A**) KLE and HEC-50 cells were treated without (Ctrl) or with 10 ng/mL TGF-β1 for different periods of time and PTEN mRNA levels were examined by RT-qPCR (normalized to GAPDH mRNA levels). (**B**) KLE and HEC-50 cells were pre-treated with vehicle (DMSO) or SB431542 (10 μM) for 1 h and then treated with 10 ng/mL TGF-β1 (T) for 24 h. PTEN protein levels were examined by Western blot (normalized to α-tubulin protein levels). Results are expressed as the mean ± SEM of at least three independent experiments and values without common letters are significantly different (*P* < 0.05).

Previous studies suggest that both KLE and HEC-50 cells have wild-type *PTEN* [25]. To examine the effect of TGF-β1 on PTEN expression, KLE and HEC-50 cells were treated with 10 ng/mL TGF-β1 for different periods of time (3, 6, 12 and 24 h). As shown in Figure 2A, treatment of KLE cells with TGF-β1 significantly down-regulated PTEN mRNA levels at 3 h and this effect was still observed after 24 h of treatment. Similarly, treatment of HEC-50 cells with TGF-β1 down-regulated PTEN mRNA levels at 6, 12 and 24 h (Figure 2A). Western blot results confirmed the suppression of PTEN protein levels by TGF-β1 at 24 h in both KLE and HEC-50 cells (Figure 2B). In addition, TGF-β1-induced decreases in PTEN protein levels were abolished by pre-treatment of KLE and HEC-50 cells with SB431542 (Figure 2B). These results indicate that TGF-β1 acts via TGF-β type I receptor to decrease PTEN mRNA and protein levels in type II endometrial cancer cells.

### Overexpression of PTEN abolishes TGF-β1-stimulated cell migration

**Figure 3:**
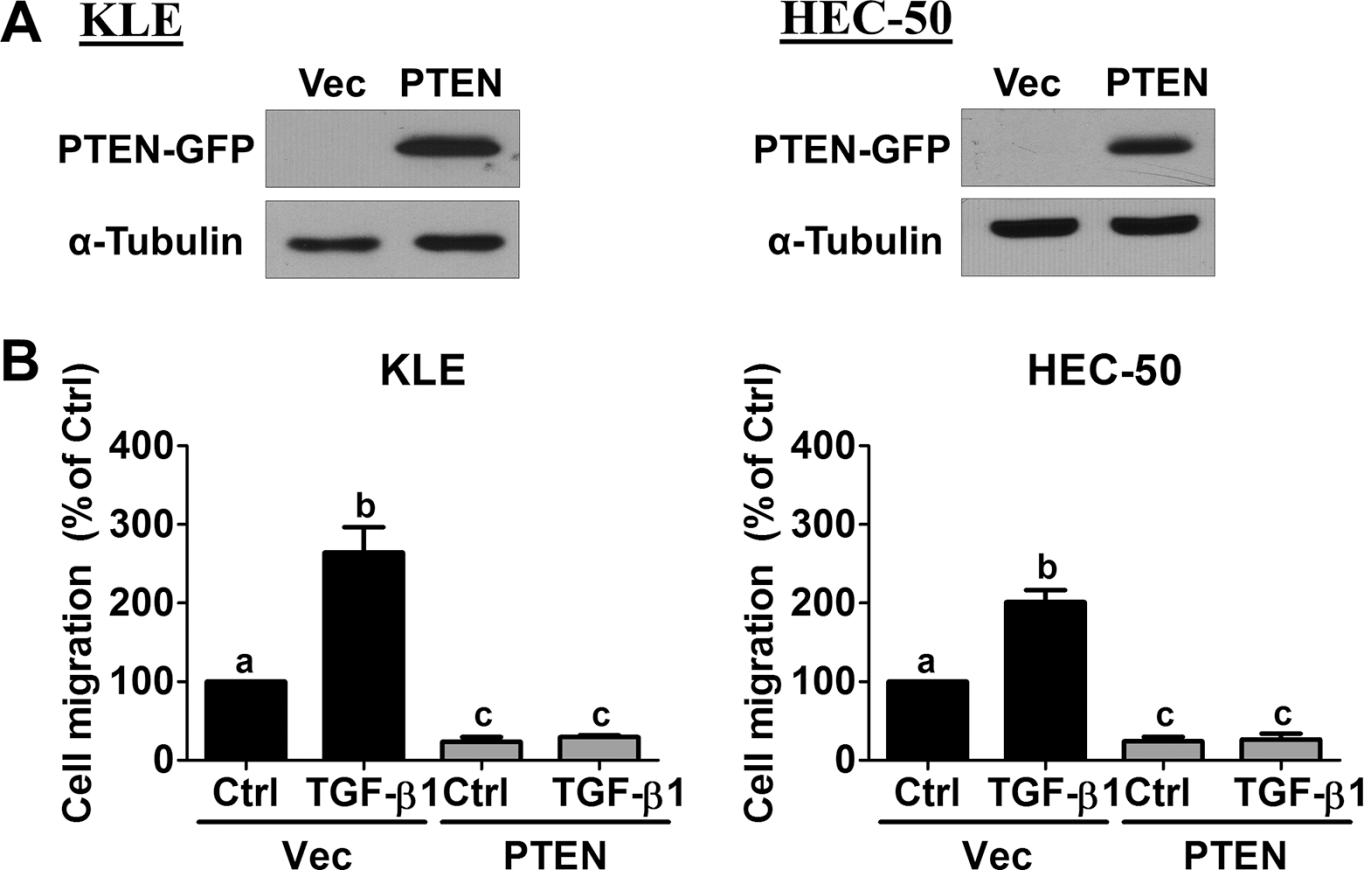
Overexpression of PTEN abolishes TGF-β1-stimulated cell migration (**A**) KLE and HEC-50 cells were transfected for 48 h with 1 μg control vector (Vec; pcDNA-GFP) or vector encoding PTEN (pcDNA-PTEN-GFP) and PTEN-GFP protein levels were examined by Western blot. (**B**) KLE and HEC-50 cells were transfected with 1 μg vector control or PTEN for 48 h and then treated without (Ctrl) or with 10 ng/mL TGF-β1 for 24 h. After treatment, the levels of cell migration were examined by the transwell migration assay (24 h). Results are expressed as the mean ± SEM of at least three independent experiments and values without common letters are significantly different (*P* < 0.05).

Forced-expression of green fluorescent protein (GFP)-tagged human PTEN was used to examine whether PTEN down-regulation contributes to the pro-migratory effects of TGF-β1 in KLE and HEC-50 cells. Western blot results confirmed ectopic expression in cells transiently transfected with PTEN-GFP vector, but not in cells transfected with empty vector (pcDNA-GFP; Figure 3A). Transwell migration assays revealed that overexpression of PTEN reduced basal and abolished TGF-β1-induced cell migration in both KLE and HEC-50 cells (Figure 3B). These results indicate that PTEN plays an important regulatory role in basal and TGF-β1-induced type II endometrial cancer cell migration.

### Inhibition of PI3K/AKT signaling attenuates TGF-β1-stimulated cell migration

**Figure 4:**
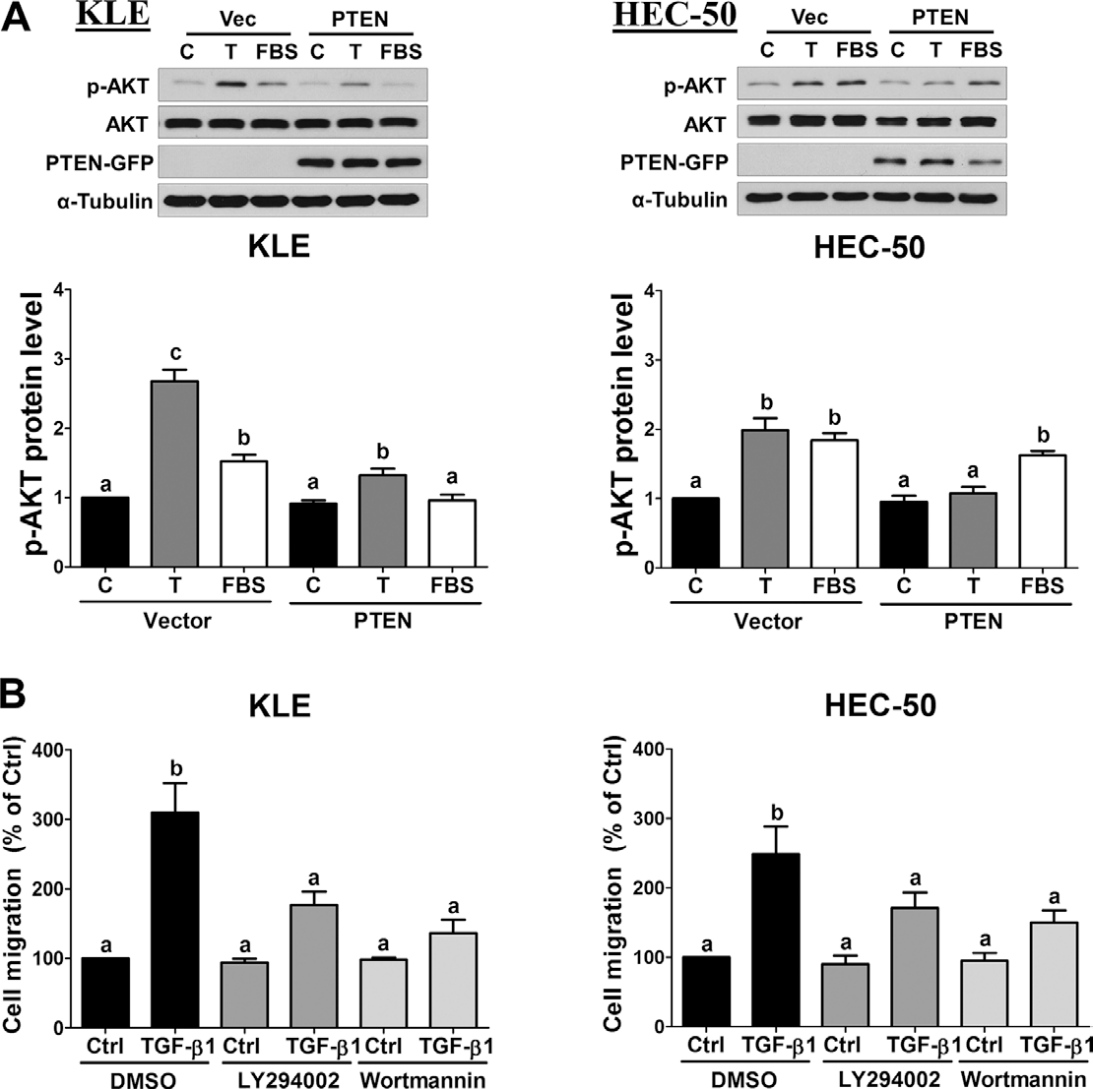
Inhibition of AKT signaling attenuates TGF-β1-stimulated cell migration (**A**) KLE and HEC-50 cells were transfected with 1 μg vector control (Vec; pcDNA-GFP) or vector encoding PTEN (pcDNA-PTEN-GFP) for 48 h and then treated without (**C**) or with 10 ng/mL TGF-β1 (T) or 10% FBS for a further 24 h. Western blot was used to confirm PTEN-GFP overexpression and to examine the levels of phosphorylated AKT (p-AKT) in relation to its total levels from the same membrane (AKT). (**B**) KLE and HEC-50 cells were pre-treated with vehicle (DMSO), LY294002 (10 μM) or Wortmannin (1 μM) for 1 h and then treated without (Ctrl) or with 10 ng/mL TGF-β1 for 24 h. After treatment, the levels of cell migration were examined by the transwell migration assay (24 h). Results are expressed as the mean ± SEM of at least three independent experiments and values without common letters are significantly different (*P* < 0.05).

Since PTEN is an important negative regulator of PI3K/AKT signaling, we next examined whether forced-expression of PTEN suppresses AKT phosphorylation/activation. As shown in Figure 4A, treatment of KLE and HEC-50 cells for 24 h with TGF-β1 increased the levels of phosphorylated AKT, and these effects were attenuated by overexpression of PTEN. Cells treated with 10% fetal bovine serum (FBS) served as a positive control. Furthermore, transwell migration assays showed that pre-treatment with two PI3K inhibitors, LY294002 and Wortmannin, attenuated TGF-β1-stimulated KLE and HEC-50 cell migration (Figure 4B). Taken together, these results suggest that TGF-β1 increases PI3K/AKT signaling which contributes, in part, to its pro-migratory effects on type II endometrial cancer cells.

### SMAD signaling is involved in the down-regulation of PTEN by TGF-β1

**Figure 5:**
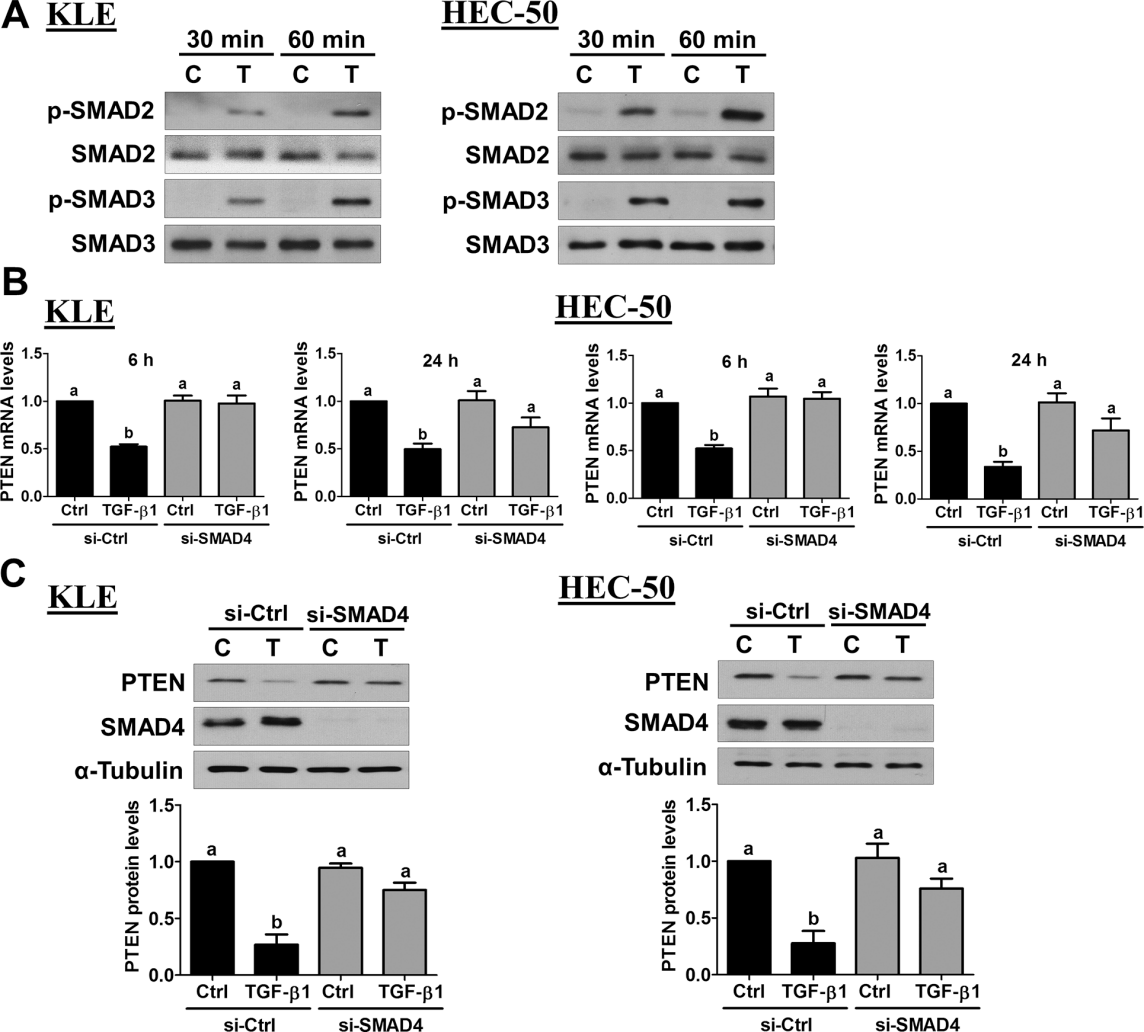
Activation of SMAD signaling is required for the down-regulation of PTEN by TGF-β1 (**A**) KLE and HEC-50 cells were treated without (**C**) or with 10 ng/mL of TGF-β1 (T) for 30 and 60 minutes. Western blot was used to examine the levels of phosphorylated SMAD2 (p-SMAD2) or SMAD3 (p-SMAD3) in relation to their total levels from the same membrane (SMAD2 and SMAD3, respectively). (**B**) KLE and HEC-50 cells were transfected for 48 h with 20 nM control siRNA (si-Ctrl) or SMAD4 siRNA (si-SMAD4) and then treated without (Ctrl) or with 10 ng/mL of TGF-β1 for 6 or 24 h. PTEN mRNA levels were examined by RT-qPCR (normalized to GAPDH mRNA levels). (**C**) KLE and HEC-50 cells were transfected for 48 h with 20 nM control or SMAD4 siRNA, treated for a further 24 h with or without 10 ng/mL of TGF-β1, and PTEN protein levels were examined by Western blot (normalized to α-tubulin protein levels). Results are expressed as the mean ± SEM of at least three independent experiments and values without common letters are significantly different (*P* < 0.05).

To date, the molecular mechanisms underlying TGF-β1-induced down-regulation of PTEN in type II endometrial cancer cells remain poorly defined. Classical TGF-β1 signaling involves the phosphorylation and activation of receptor-regulated SMAD2/3, which subsequently bind to common SMAD4 to form complexes that regulate transcription. To investigate the involvement of canonical SMAD2/3-SMAD4 signaling, we first used Western blot to examine SMAD2/3 phosphorylation/activation. As shown in Figure 5A, TGF-β1 treatment significantly increased the phosphorylation of SMAD2 and SMAD3 in KLE and HEC-50 cells. To directly examine the involvement of SMAD signaling, we measured the effects of TGF-β1 on PTEN mRNA levels at early (6 h) and late (24 h) time-points following siRNA-mediated knockdown of common SMAD4. As shown in Figure 5B, knockdown of SMAD4 abolished the suppressive effects of TGF-β1 on PTEN mRNA at 6 h and significantly attenuated its effects at 24 h in both KLE and HEC-50 cells. Western blot analysis showed that SMAD4 knockdown attenuated the suppression of PTEN protein by TGF-β1 at 24 h in both KLE and HEC-50 cells (Figure 5C; TGF-β1 did not suppress PTEN protein at 6 h in either cell line). Likewise, combined knockdown of SMAD2 and SMAD3 inhibited the down-regulation of PTEN protein by TGF-β1 in KLE and HEC-50 cells (Supplementary Figure S1). Together, these results suggest that classical SMAD2/3-SMAD4 signaling contributes to the down-regulation of PTEN by TGF-β1 in type II endometrial cancer cells.

### MEK-ERK1/2 signaling is required for TGF-β1-induced down-regulation of PTEN

**Figure 6:**
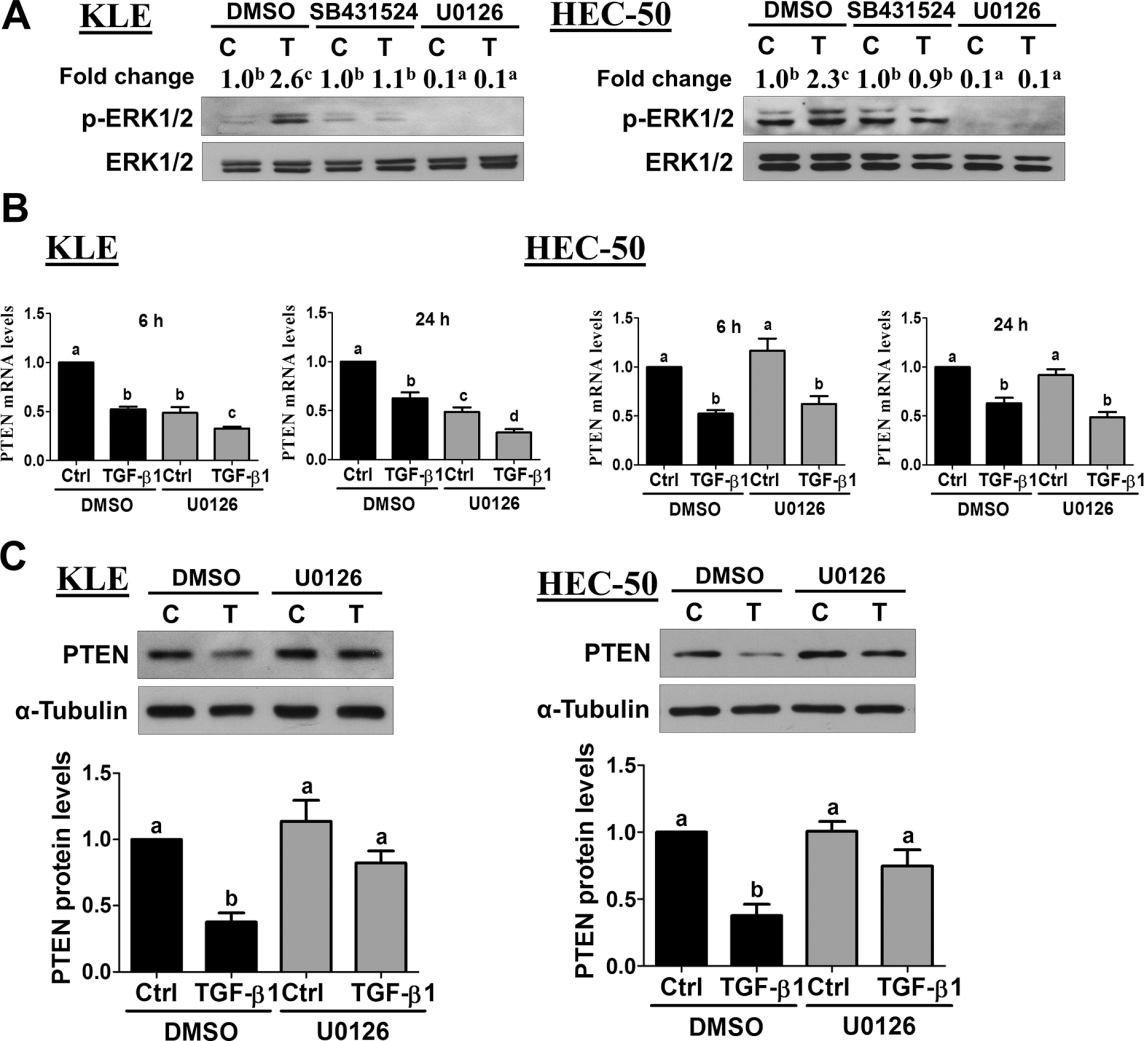
Activation of ERK1/2 signaling is required for the down-regulation of PTEN by TGF-β1 (**A**) KLE and HEC-50 cells were pre-treated for 1 h with vehicle (DMSO), SB431542 (10 μM) or U0126 (10 μM) and then treated without (**C**) or with 10 ng/mL TGF-β1 (T) for 10 min. Western blot was used to examine the levels of phosphorylated ERK1/2 (p-ERK1/2) in relation to its total levels from the same membrane (ERK1/2). Summarized quantitative results are displayed numerically as the mean fold change with superscripted letters indicating statistical significance as described below. (**B**) KLE and HEC-50 cells were pre-treated for 1 h with or without U0126 (10 μM) and then treated without (Ctrl) or with 10 ng/mL TGF-β1 for 6 or 24 h. PTEN mRNA levels were examined by RT-qPCR (normalized to GAPDH mRNA levels). (C) KLE and HEC-50 cells were pre-treated for 1 h with U0126 (10 μM), treated for a further 24 h with 10 ng/mL TGF-β1, and PTEN protein levels were examined by Western blot (normalized to α-tubulin protein levels). Results are expressed as the mean ± SEM of at least three independent experiments and values without common letters are significantly different (*P* < 0.05).

Accumulating evidence suggests that ERK1/2 signaling is closely associated with the TGF-β1 pathway in tumorigenesis [26]. In endometrial cancer, gene expression profiling suggests that MEK-ERK1/2 and TGF-β1 gene networks are associated with a high risk of recurrence [17]. Therefore, we investigated whether MEK-ERK1/2 signaling was involved in the down-regulation of PTEN by TGF-β1. First, we used Western blot to examine the effects of TGF-β1 on ERK1/2 phosphorylation/activation. As shown in Figure 6A, TGF-β1 treatment increased ERK1/2 phosphorylation in both KLE and HEC-50 cells. These increases in ERK1/2 phosphorylation were abolished by pre-treatment with the TGF-β type I receptor inhibitor SB431542 (Figure 6A). In addition, pre-treatment with the MEK inhibitor U0126 not only decreased basal levels of phosphorylated ERK1/2 but also abolished TGF-β1-induced ERK1/2 phosphorylation (Figure 6A). Next, we used U0126 to examine the role of MEK-ERK1/2 signaling in the suppression of PTEN by TGF-β1 in KLE and HEC-50 cells. As shown in Figure 6B, pre-treatment with U0126 failed to inhibit the down-regulation of PTEN mRNA at both early (6 h) and late (24 h) time-points. Interestingly, Western blot analysis showed that U0126 pre-treatment attenuated the suppression of PTEN protein by TGF-β1 at 24 h in both KLE and HEC-50 cells (Figure 6C). These results suggest that TGF-β1-induced MEK-ERK1/2 signaling is involved post-transcriptionally in the suppression of PTEN protein, whereas it is not involved in the down-regulation of PTEN mRNA in type II endometrial cancer cells.

## DISCUSSION

Endometrial cancers are traditionally classified into two types defined by Bokhman [2]. Type I endometrial cancers are estrogen-dependent, non-metastatic, and associated with favorable prognosis, whereas type II endometrial tumors are estrogen-independent, highly invasive and more lethal [3, 4]. It has been shown that in type I endometrial cancers, the levels of SMAD2 phosphorylation are weak or undetectable, and the expression levels of type I and type II TGF-β receptors are decreased. Moreover, primary cultures of type I endometrial cancers do not respond to TGF-β1-mediated growth inhibition [11]. These results suggest that classical TGF-β1 signaling may be deregulated at early stages in the development of type I endometrial cancers, leading to their escape from normal modes of growth control. Moreover, TGF-β1 can promote the invasiveness of type I endometrial cancer cell lines Ishikawa [27] and RL-95-2 [17]. As for type II endometrial cancers, the effects of TGF-β1 on their growth are inconsistent. TGF-β1 has been found to inhibit the growth of HEC-1A cells [18], whereas it stimulates the growth of HEC-1B cells [27]. Regarding the effects of TGF-β1 on type II endometrial cancer cell motility, our results show that TGF-β1 can enhance cell migration via both SMAD-dependent and -independent signaling. Since cell invasion is a complex process involving not only migration, but also adhesion and proteolysis, future studies examining the effects of TGF-β1 on these cellular functions will be of interest. Together, these results suggest that the invasive and metastatic capacity of endometrial cancer cells is likely enhanced by the core components of the TGF-β1 pathway. This is in keeping with the TGF-β1 paradox theory wherein TGF-β loses its anti-growth effects and promotes cancer progression at a late stage [28].

To date, various approaches targeting the TGF-β1 pathway are under preclinical or clinical investigation and have been shown to exhibit anti-tumor activity [29]. However, only a handful of studies emphasize the therapeutic potential of abrogating TGF-β1 signaling in endometrial cancer. One study shows that ectopic expression of dominant-negative TGF-β type II receptor significantly inhibits lung metastases in a mouse xenograft model of HEC-1A endometrial cancer cells [18]. Mechanistically, many pro-oncogenic responses to TGF-β1 require the participation of SMAD-independent pathways [30], such as the ERK1/2 signaling pathway, which has a sophisticated and intimate relationship with the TGF-β1 system in regulating tumorigenesis [26]. Indeed, gene expression studies have implicated MEK-ERK1/2 and TGF-β1 gene networks in endometrial cancer recurrence [17]. The effects of TGF-β1 on ERK1/2 signaling vary considerably among cell types and the exact mechanisms of this link remain largely unclarified. MEK-ERK1/2 signaling may be activated directly by TGF-β1 [31] or indirectly via transcription factors [32]. In the present study, ERK1/2 signaling is directly activated by TGF-β1 and contributes to the down-regulation of PTEN in type II endometrial cancer cells. Recent studies suggest that activation of ERK1/2 signaling may convert the growth inhibitory effects of TGF-β1 to more pro-oncogenic effects, and cooperation between the TGF-β1 pathway and activated ERK1/2 signaling is essential for the invasive phenotype in different types of human cancer [26, 29, 33]. Thus, inhibition of ERK1/2 signaling could block the tumor-promoting roles of TGF-β1 signaling while retaining its tumor suppressive effects in endometrial cancer. Evidence to date suggests that ERK1/2 and SMAD signaling can interact at multiple levels. For example, phosphorylation of the linker region of SMADs by ERK1/2 has been shown to modulate their downstream effects [32, 34]. ERK1/2 signaling can also cooperate with SMAD complexes by regulating transcriptional co-factors at target gene promoters [35]. Interestingly, our results suggest that the suppression of PTEN mRNA by TGF-β1 appears to be solely mediated by SMAD2/3-SMAD4 signalling in type II endometrial cancer cells. In contrast, the suppressive effects of TGF-β1-MEK-ERK1/2 signaling on PTEN appear to be exerted at the protein level since MEK inhibition did not affect the down-regulation of PTEN mRNA. Accumulating evidence suggests that the stability, activity and cellular localization of PTEN can be regulated by phosphorylation of residues in its C-terminal tail [36], and future studies investigating how TGF-β1-MEK-ERK1/2 signaling regulates PTEN protein levels will be of great interest.

*PTEN* mutations are common in type I endometrial cancers (50–80%), especially in tumors of endometrioid histology [3, 20]. While *PTEN* mutations are rare in type II endometrial cancers, PTEN protein loss is frequently detected [21, 22]. These results strongly indicate that loss of PTEN expression in type II endometrial cancers may occur via transcriptional or post-translational mechanisms. In liver cancer cells, TGF-β1 down-regulates PTEN expression by accelerating the turnover rate of PTEN mRNA and increasing ubiquitin-proteasome-mediated PTEN protein degradation, without affecting its transcription [37]. In KLE cells, PTEN protein degradation has been shown to be increased by TGF-β1 [23]. The present study provides evidence that, in addition to its post-translational regulation, the expression of PTEN can be transcriptionally regulated by TGF-β1 in type II endometrial cancers.

In endometrial cancer, activation of AKT signaling is observed in approximately 60% of patients with recurrent/metastatic disease [38]. In addition, aberrant activation of PI3K/AKT signaling is associated with a more aggressive pathology and poorer prognosis, irrespective of endometrial cancer subtype [39]. Dysregulated PTEN and subsequent hyper-activation of the PI3K/AKT pathway has been implicated in cell motility [40, 41], and regulation of PTEN/PI3K/AKT activity has been implicated in tumorigenesis and anticancer therapy resistance [42]. In the present study, we showed that overexpression of PTEN attenuated TGF-β1-induced activation of AKT and cell migration in type II endometrial cancer cells. In addition, we showed that inhibition of PI3K attenuated TGF-β1-stimulated cell migration. Consistent with previous findings, our results further support that PTEN acts as a tumor suppressor by inhibiting AKT activation, which plays important roles in regulating the progression of type II endometrial cancer. In a phase II study for endometrial cancer, the MEK inhibitor Selumetinib has demonstrated only limited single-agent activity [43]. This could be attributed to the presence of other activated oncogenic signaling pathways, such as PI3K/AKT, Wnt/β-catenin and epidermal growth factor receptor/HER2 [44]. Therefore, simultaneous inhibition of multiple oncogenic pathways may generate better therapeutic results. Indeed, to test this hypothesis, a clinical trial for endometrial cancer is ongoing to evaluate combined treatment with the MEK inhibitor Trametinib and the AKT inhibitor GSK2141795 (ClinicalTrials.gov Identifier: NCT01935973).

**Figure 7:**
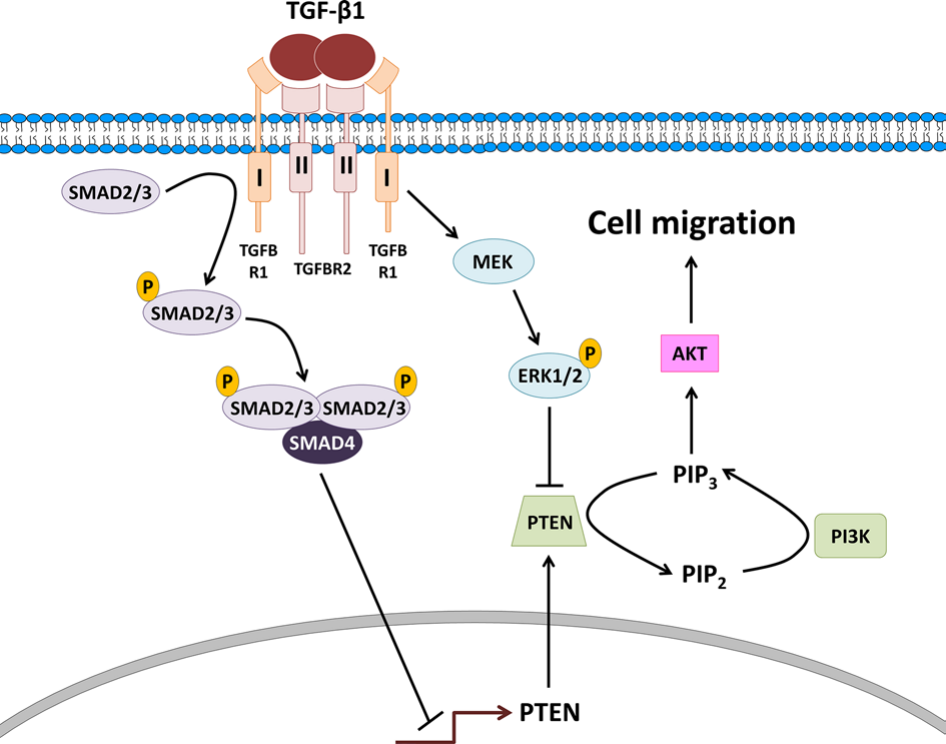
Proposed model for the actions of TGF-β1 on PTEN, PI3K-AKT signaling and cell migration in type II endometrial cancer cells TGF-β1 binds to a complex of type I and II receptors leading to the phosphorylation/activation of receptor-regulated SMAD2/3 which bind to common SMAD4 and translocate into the nucleus to decrease the transcription of *PTEN*. In parallel, the ligand-receptor complex subsequently activates MEK leading to the phosphorylation/activation of ERK1/2 which acts post-transcriptionally to suppress PTEN protein. The down-regulation of PTEN enhances PI3K-AKT signaling which is an essential mediator of TGF-β1-induced type II endometrial cancer cell migration.

In summary, we demonstrate that TGF-β1 down-regulates PTEN at the transcriptional level via SMAD2/3-SMAD4 signaling and post-transcriptionally via MEK-ERK1/2 signaling. Reduced levels of PTEN enhance PI3K-AKT signaling which is an essential mediator of TGF-β1-induced type II endometrial cancer cell migration (Figure 7). Our study provides important insights into the molecular mechanisms by which TGF-β1 may contribute to the metastasis of type II endometrial cancers.

## MATERIALS AND METHODS

### Cell culture

The KLE human endometrial cancer cell line was purchased from the American Type Culture Collection (Manassas, VA). The HEC-50, also known as HEC-50B, human endometrial cancer cell line was obtained from the OVCARE Cell Bank (Vancouver, BC). Both cell lines were cultured in Dulbecco's Modified Eagle Medium/Nutrient Mixture F-12 (Gibco, Life Technologies) supplemented with 10% fetal bovine serum (Hyclone Laboratories Inc.). Cultures were maintained at 37°C in a humidified atmosphere of 5% CO_2_ in air.

### Antibodies and reagents

The rabbit polyclonal antibodies used in this study were: SMAD4 (#9515, Cell Signaling Technology), phospho-ERK1/2 (Thr202/Tyr204; #9101, Cell Signaling Technology), ERK1/2 (#9102, Cell Signaling Technology), phospho-AKT (Ser473; #9271, Cell Signaling Technology), AKT (#9271, Cell Signaling Technology). The rabbit monoclonal antibodies used in this study were: PTEN (#9559, Cell Signaling Technology), phospho-SMAD2 (Ser465/467; 138D4, Cell Signaling Technology), phospho-SMAD3 (Ser423/425; C25A9, Cell Signaling Technology), and SMAD3 (C67H9, Cell Signaling Technology). The mouse monoclonal antibodies used were: SMAD2 (L16D3, Cell Signaling Technology) and α-tubulin (B-5-1-2, Santa Cruz). Horseradish peroxidase-conjugated goat anti-mouse IgG and goat anti-rabbit IgG were obtained from Bio-Rad Laboratories (Hercules, CA). SB431542 and U0126 were purchased from Sigma-Aldrich (Oakville, ON). TGF-β1 was obtained from R&D Systems (Minneapolis, MN).

### Transwell migration assays

Migration assays were performed in Boyden chambers. Cell culture inserts (24-well, pore size 8 μm; BD Biosciences, Mississauga, ON) were seeded with 1 × 10^5^ cells in 250 μL of medium with 0.1% FBS. Un-coated inserts were used for migration assay. Medium with 10% FBS (750 μL) was added to the lower chamber and served as a chemotactic agent. After incubation for 24 h, non-migrating cells were wiped from the upper side of the membrane and cells on the lower side were fixed in cold methanol and air dried. Cell nuclei were stained with crystal violet and counted. Each individual experiment had triplicate inserts and five microscopic fields were counted per insert.

### Reverse transcription quantitative real-time PCR (RT-qPCR)

Total RNA was extracted using TRIzol reagent (Invitrogen, Life Technologies, Burlington, ON) in accordance with the manufacturer's instructions. Reverse transcription was performed with 2 μg RNA, random primers and M-MLV reverse transcriptase (Promega, Madison, WI). The primers used for SYBR Green RT-qPCR were: PTEN, 5′-CGA ACT GGT GTA ATG ATA TGT -3′ (forward) and 5′-CAT GAA CTT GTC TTC CCG T -3′ (reverse) and GAPDH, 5′-GAG TCA ACG GAT TTG GTC GT-3′ (forward) and 5′- GAC AAG CTT CCC GTT CTC AG-3′ (reverse). RT-qPCR was performed using an Applied Biosystems 7300 Real-Time PCR System equipped with 96-well optical reaction plates. The specificity of each assay was validated by melting curve analysis and agarose gel electrophoresis of the PCR products. Assay performance was validated by assessing amplification efficiencies by means of calibration curves, and ensuring that the plot of log input amount versus ΔCq has a slope < |0.1|. At least three separate experiments were performed and each sample was assayed in triplicate. A mean value of the triplicates was used for the determination of relative mRNA levels by the comparative Cq method with GAPDH as the reference gene and using the formula 2^−ΔΔCq^.

### Western blot

Cells were lysed in ice cold lysis buffer (Cell Signaling Technology) with added protease inhibitor cocktail (Sigma-Aldrich). Extracts were centrifuged at 20,000 × g for 10 min at 4°C and supernatant protein concentrations were determined using the DC Protein Assay (Bio-Rad Laboratories). Equal amounts of protein were separated by SDS polyacrylamide gel electrophoresis and transferred onto PVDF membranes. After blocking for 1 h with 5% non-fat dry milk in Tris-buffered saline (TBS), the membranes were incubated overnight at 4°C with primary antibodies that were diluted 1000-fold in 5% non-fat milk-TBS. Following primary antibody incubation, the membranes were incubated with the appropriate HRP-conjugated secondary antibody. Immunoreactive bands were detected using enhanced chemiluminescent substrate or SuperSignal West Femto chemiluminescent substrate (Thermo Fisher) and X-ray film. Membranes were stripped with stripping buffer (50 mM Tris-HCl pH 7.6, 10 mM β-mercaptoethanol, and 1% SDS) at 50°C for 30 minutes and reprobed with anti-α-tubulin, anti-AKT, anti-SMAD2, anti-SMAD3 or anti-ERK1/2 for normalization. Immunoreactive band intensities were quantified by densitometry using Scion Image software (Scion Corp, Frederick, MD) and normalized to those of the relevant loading control.

### Plasmid constructs and transfection

pcDNA-GFP and pcDNA-PTEN-GFP were generously provided by Dr. Alonzo H. Ross (University of Massachusetts Medical School, Worcester, MA, USA). Cells at 60% confluence were transfected for 48 h with the pcDNA-GFP or pcDNA-PTEN-GFP vector using Lipofectamine 3000 (Life Technologies) according to the manufacturer's instructions.

### Small interfering RNA (siRNA) transfection

To knock down endogenous SMAD4, 60% confluent cells were transfected for 48 h with 20 nM ON-TARGETplus SMART pool siRNA targeting human SMAD4 (Dharmacon, Lafayette, CO) using Lipofectamine RNAiMAX (Invitrogen, Life Technologies). ON-TARGETplus Non-targeting pool siRNA (Dharmacon) was used as the control.

### Statistical analysis

The results are presented as the mean ± SEM of at least three independent experiments. For experiments involving only two groups, the results were analysed by a two-sample *t-test* assuming unequal variances using Excel. Multiple group comparisons were analysed by one-way ANOVA followed by Student-Newman-Keuls tests using PRISM software (GraphPad Software). The means were considered significantly different if *P* < 0.05 and are indicated by different letters.

## SUPPLEMENTARY MATERIAL FIGURES


