# Supplementary material for: TGF-β1 stimulates migration of type II endometrial cancer cells by down-regulating PTEN via activation of SMAD and ERK1/2 signaling pathways
Source: Oncotarget. 2016 Aug 16;7(38):61262–72. doi: 10.18632/oncotarget.11311 (PMC5308649; doi:10.18632/oncotarget.11311)
Supplement: Supplementary file 1 [file oncotarget-07-61262-s001.pdf]

TGF-β1 stimulates migration of type II endometrial cancer cells by down-regulating PTEN via activation of SMAD and ERK1/2 signaling pathways

Supplementary Materials

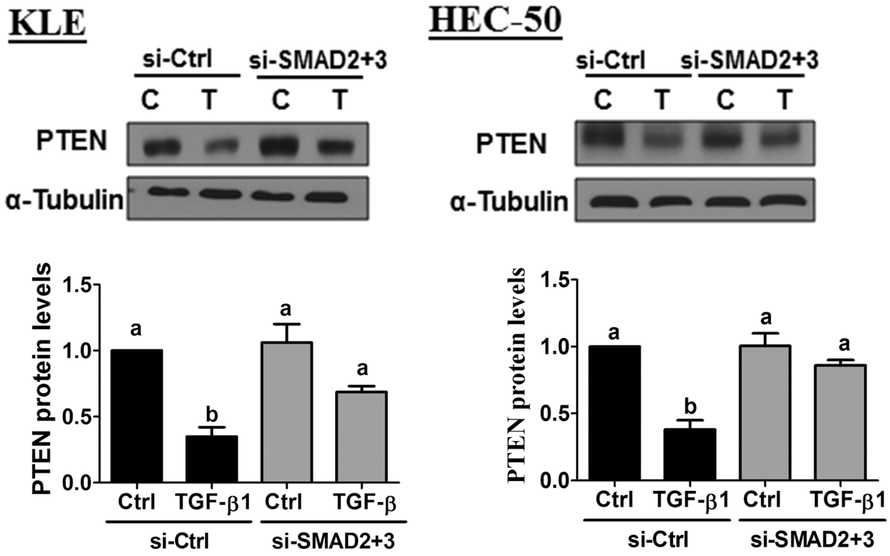

Supplementary Figure S1: KLE and HEC-50 cells were transfected for 48 h with 50 nM control siRNA (si-Ctrl) or combined SMAD2 and SMAD3 siRNA (si-SMAD2+3), treated for a further 24 h without (C) or with 10 ng/mL TGF-β1 (T), and PTEN protein levels were examined by Western blot (normalized to α-tubulin protein levels). Results are expressed as the mean ± SEM of two independent experiments and values without common letters are significantly different ($P < 0.05$).
